# Distal femoral fractures: periprosthetic fractures have four times more complications than non-periprosthetic fractures and cerclage should be avoided: retrospective analysis of 206 patients

**DOI:** 10.1186/s10195-024-00782-2

**Published:** 2024-09-03

**Authors:** Martin Direder, Cornelia Naß, Julian Ramin Andresen, Theresa Dannenmann, Florian Bur, Stefan Hajdu, Thomas Haider

**Affiliations:** Department of Orthopedics and Trauma-Surgery, Medical University of Vienna, Lazarettgasse 14, 1090 Vienna, Austria

**Keywords:** Distal femoral fracture, Plate osteosynthesis, Cerclage, Complication rate

## Abstract

**Background:**

Distal femoral fractures account for less than 1% of all fractures. The therapy of choice is usually surgical stabilization. Despite advances in implant development over the past few years, complication rate remains comparatively high. The aim of this study is to analyze our results with plate fixation of distal femoral fractures with a focus on complication and fracture healing rates.

**Methods:**

In this retrospective cohort study, patients (> 18 years) with distal femoral fractures treated at an urban level I trauma center between 2015 and 2022 were analyzed.

**Results:**

In total, 206 patients (167 female, 39 male) with an average age of 75 (SD 16) years were diagnosed with a fracture of the distal femur. One hundred fourteen of these patients were treated surgically by means of plate osteosynthesis. In 13 cases (11.41%), a revision procedure had to be performed. The indication for surgical revision was mechanical failure in eight cases (7.02%) and septic complication in five cases (4.39%). Periprosthetic fractures were more likely to cause complications overall (19.6% versus 4.76%) and further included all documented septic complications. The analysis of modifiable surgical factors in the context of plate osteosynthesis showed higher complication rates for cerclage in the fracture area compared with plate-only stabilizations (44.44% versus 22.22%).

**Conclusions:**

The data show an increased amount of revisions and a significantly higher number of septic complications in the treatment of periprosthetic fractures of the distal femur compared with non-periprosthetic fractures. The detected combination of plates together with cerclage was associated with higher complication rates.

*Level of evidence* Level III retrospective comparative study.

**Supplementary Information:**

The online version contains supplementary material available at 10.1186/s10195-024-00782-2.

## Introduction

Fractures of the distal femur are a comparatively rare bone injury (approximately 1:250) [1]. The risk of a periprosthetic fracture of the distal femur following total knee arthroplasty ranges between 0.9% and 1.6% [2]. Previous scientific work showed an increased incidence of distal femoral fractures in older women and young men [3]. This is a result of declining bone quality in older patients, especially women, and the frequent occurrence of these fractures in young patients after high-speed trauma, such as motorcycle accidents [4]. Additional typical risk factors include osteoporosis, nicotine consumption, diabetes, vascular diseases, advanced age, malignant diseases, rheumatism, and nonsteroidal anti-inflammatory drug (NSAID) and steroid use [2, 5, 6]. Distal femoral fractures can be subdivided according to the “Arbeitsgemeinschaft für Osteosynthesefragen Foundation/Orthopaedic Trauma Association” (AO/OTA) classification system, which classifies the fracture according to its localization, articular involvement, and extent of comminution [7]. Periprosthetic fractures on the other side can be classified using the Unified Classification System (UCS), a general system to categorize periprosthetic fractures according to the fracture area, the involvement of the prosthesis, and the bone constellation [8]. In most cases, surgical stabilization is the treatment of choice as it has been shown to be associated with higher union rates, improved function, and bone alignment [9, 10]. Surgical treatment options range from different plate types to intermedullary nails and recently even a combination of both treatments [11]. An extensive metaanalysis focusing on the complication rates following internal fixation for distal femoral fractures by plate or nail uncovered nonunion as main complication, followed by mechanical failure, infections, and other union complications [12]. To support proper fracture fixation, additional tools such as cerclages are occasionally used, but an increased complication rate, especially nonunion, infection, and mechanical failure were previously associated with their use [13–17]. The distance between the first screws on each side of a fracture is termed the working length [18]. Biomechanical analysis tried to identify the relation between working length and fracture healing [19–22]. Nonetheless, the optimal plate osteosynthesis configuration including screw and cerclage placement still requires more research. The aim of this study is to analyze distal femoral fractures treated at our level 1 trauma center between 2015 and 2022, to study surgical variables and their influence on complication and revision rates.

## Materials and methods

This retrospective cohort study was approved by the local ethics committee (approval number 1236/2023). Systematic screening on the admission data of patients aged 18 years or older with fractures of the distal femur between January 2015 and December 2022 at our urban level I trauma center was performed. Only closed fractures of the distal femur were recorded in this study. Gathered patient data included sex, age, trauma mechanism, periprosthetic or non-periprosthetic fracture, risk factors such as nicotine, diabetes, vascular disease, malignant disease, rheumatism, use of NSAIDs or steroids, osteoporosis, type of operation/treatment, surgical revision, type of revision, union, patient mobility, operation technique, distal fixation, additional fixation, and length of follow-up. Non-periprosthetic fractures were classified according to the AO classification, while periprosthetic fractures were classified according to the UCS classification, on the basis of the first appropriate X-ray taken upon injury [7, 8]. Fracture and plate measurements were performed on the first appropriate radiologic control after surgery. Union or nonunion was documented on the basis of signs for radiographic union at the last accessible X-ray of at least 6 months after surgery. In addition, the Parker mobility score was calculated on the basis of available data. Patients with incomplete datasets were excluded. Conservative treatment and primary surgery performed at another hospital were exclusion criteria for subset data investigations. Five independent observers performed the data acquisition. Four observers were orthopedic trauma surgery residents in their first of training (PGY1), and one observer was a medical student in his final year at university. Data acquisition was overseen by a fellowship-trained orthopedic surgeon with a special interest in orthopedic trauma. Schematic drawings of the AO classification and of the measured radiologic parameters with precise information regarding correct measurement were available during the data acquisition for each observer. Correct measurement was subsequently confirmed for each observer using random samples. No considerable differences in measurement performance were recognized among all observers.

### Statistical analysis

Statistical analysis was performed using R (version 4.0.3; The R Foundation, Vienna, Austria), R-studio as well as GraphPad Prism 5 (GraphPad Software Inc., La Jolla, CA, USA). Student’s *t*-test was used for comparison of normal distributed mean values, and the Mann–Whitney *U*-test and the Kruskal–Wallis test were applied for non-normally distributed values. Fisher’s exact test was performed to compare characteristics with nominal or ordinal scales. Tukey’s range test was used for multiple test correction. *p*-Value < 0.05 was considered statistically significant.

## Results

### Demographics

**Fig. 1:**
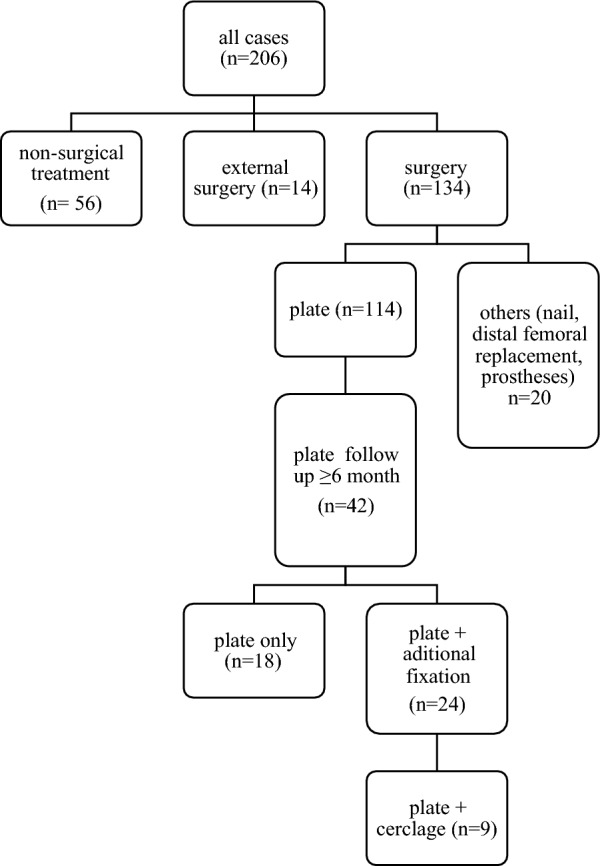
Flow diagram of the study population

**Fig. 2:**
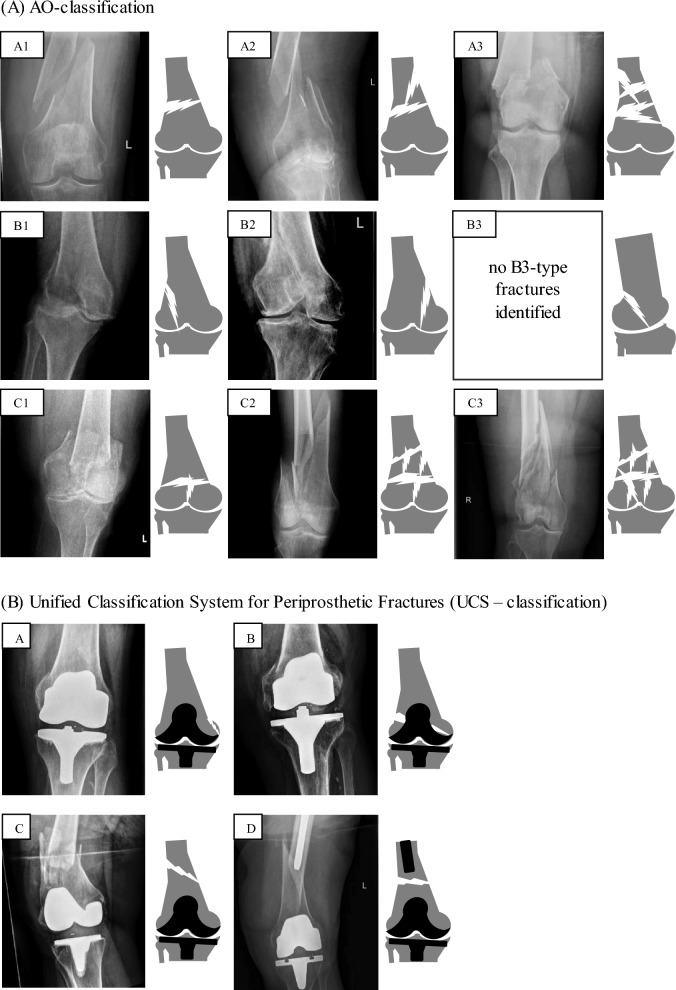
Fracture classifications. Schematic representation of fracture classifications with original X-rays of recorded patients. **A** AO classification: A1, simple fracture; A2, metaphyseal wedge-shaped fracture; A3, metaphyseal complex fracture; B1, lateral condyle sagittal fracture; B2, medial condyle sagittal fracture; B3, fracture of the anterior part of the femoral condyle; C1, simple intraarticular and metaphyseal fracture; C2, simple intraarticular and comminuted metaphyseal fracture; C3, comminuted fracture; **B** UCS classification: A, periarticular; B, bed of the implant; C, distant of the implant; D, dividing the bone between two implants; E and F not shown as irrelevant for this fracture area

**Fig. 3:**
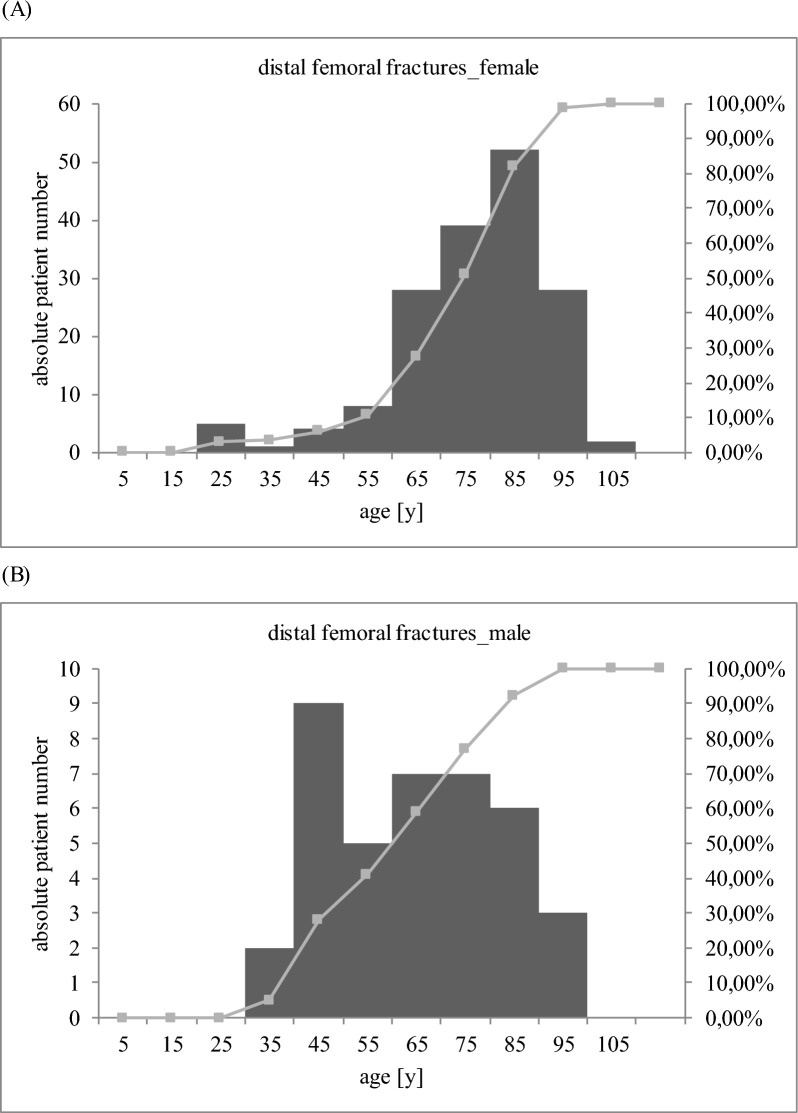
Histogram showing sex-specific, age-related patient distribution

Between 2015 and 2022, a total of 206 patients with a mean age of 75 years (SD 16 years) were diagnosed with distal femoral fracture at our level I trauma center (Fig. 1; Table 1). One hundred sixty-seven patients were female (81.07%), and the mean available follow-up was 7 months. With 90% prevalence, a simple fall was the main trauma mechanism, followed by traffic accident (4%) and rotational trauma (3%). One hundred thirty-four patients (65%) received surgical treatment at our department, 56 patients (27%) were treated conservatively, and 16 patients (8%) underwent surgical treatment at an external hospital. There were more non-periprosthetic (62%) than periprosthetic distal femoral fractures (38%). According to the AO classification, most of the non-periprosthetic fractures were A type fractures (A1: 32%; A2: 10%; A3: 7%) followed by C-type fractures. In case of periprosthetic fractures, the majority was classified as C-type fractures (23%) according to the UCS classification (Table 1; Fig. 2). The most frequently recorded risk factor was advanced age, defined as older than 65 years of age (78%), followed by osteoporosis (32%), vascular disease (29%), and diabetes (28%). Taking age and sex together, there was an increased incidence of fractures in women aged above 80 years, whereas in men the age peak occurred at a younger age with a mean of 65 years (Fig. 3a, b).

**Table 1:** General demographics and patient characteristics

Characteristics		All patients	
*n*		**206**	
Mean age (years)		75 (± 16)	
Female (%)		167	81.07%
Follow-up (months)		7	
Trauma mechanism	Simple fall	179	89.95%
	Traffic accident	8	4.02%
	Distorsion	6	3.02%
	Fall from great height	4	2.01%
	Attack	2	1.01%
	N/a	7	Valid percentage only
Fracture type	Periprosthetic	79	38.35%
	Non-periprosthetic	127	61.65%
AO classification (non-periprosthetic)	A1	66	32.04%
	A2	21	10.19%
	A3	14	6.80%
	B1	5	2.43%
	B2	3	1.46%
	B3	0	0.00%
	C1	5	2.43%
	C2	9	4.37%
	C3	4	1.94%
UCS classification (periprosthetic)	A	2	0.97%
	B	25	12.14%
	C	48	23.30%
	D	4	1.94%
	E	0	0.00%
	F	0	0.00%
Operation	Yes	134	65.05%
	No	56	27.18%
	External	16	7.77%
Nicotine	Yes	37	25.00%
	No	111	75.00%
	N/a	58	Valid percentage only
Diabetes	Yes	52	27.96%
	No	134	72.04%
	N/a	20	Valid percentage only
Vascular disease	Yes	54	29.03%
	No	132	70.97%
	N/a	20	Valid percentage only
Advanced age	Yes	160	77.67%
	No	46	22.33%
	N/a	0	Valid percentage only
Malignant disease	Yes	28	14.81%
	No	161	85.19%
	N/a	17	Valid percentage only
Rheumatism	Yes	1	0.53%
	No	189	99.47%
	N/a	16	Valid percentage only
NSAID	Yes	36	20.22%
	No	142	79.78%
	N/a	28	Valid percentage only
Steroid use	Yes	9	5.06%
	No	169	94.94%
	N/a	28	Valid percentage only
Osteoporosis	Yes	59	31.72%
	No	127	68.28%
	N/a	20	Valid percentage only

*N/a* not available

### Comparison of periprosthetic and native distal femur fractures treated with plate osteosynthesis

Of all 134 patients who received surgical treatment at our department, 114 patients (85%) were treated with open reduction and internal fixation by plate (Fig. 1; Table 2). In the next section, all patients who were treated with plate fixation are analyzed in more detail. Frequency of periprosthetic and native distal femoral fractures was comparable within this group (51 versus 63). Patients with periprosthetic fractures were significantly older compared with patients with native distal femoral fractures (mean 82 versus 70 years, *p* =  < 0.0001). We did not find a significant difference in sex distribution between both groups (*p* = 0.4938). Mean follow-up of all patients was 7 months. Evaluation of the trauma mechanism revealed significant differences between the two groups as 51 patients (100%) of included periprosthetic fractures were induced by simple fall (*p* = 0.0011) and about 8% of all non-periprosthetic fractures were caused by a traffic accident (*p* = 0.0206). Distal fixation in the presence of a total knee arthroplasty can be challenging. However, no significant difference in total number of screws distal to the fracture between periprosthetic and non-periprosthetic fractures was detected. However, a significantly lower number of bicortical screws was detected in the non-periprosthetic group (3.7 [± 2,5] versus 4.5 [± 1,7]; *p* = 0.0405). No difference in the requirement of additional fixation was detected. A significant higher number of operations without second intervention was detected in the group of non-periprosthetic fractures (95% versus 80%, *p* = 0.0175). Furthermore, a significant increase of septic revisions was prevalent in the periprosthetic group (10% versus 0%, *p* = 0.0160).

**Table 2:** Periprosthetic versus non-periprosthetic fractures: complication and trauma characteristics

Characteristic		All patients		Periprosthetic		Non-periprosthetic		*p*-Value
*n*		114		51		63		
Mean age (years)		76 (± 16)		82 (± 17)		70 (± 5)		** < 0.0001**
Female (%)		94	82.46%	44	86.27%	50	79.37%	0.4938
Follow-up (months)		7		8		7		0.6732
Trauma mechanism	Simple fall	102	90.27%	51	100.00%	51	82.26%	**0.0011**
	Traffic accident	5	4.42%	0	0.00%	5	8.06%	**0.0206**
	Distorsion	2	1.77%	0	0.00%	2	3.23%	1
	Fall from great height	3	2.65%	0	0.00%	3	4.84%	0.1397
	Attack	1	0.88%	0	0.00%	1	1.61%	1
	N/a	1	Valid percentage only	0	Valid percentage only	1	Valid percentage only	
Operation technique	Open	108	94.74%	48	94.12%	60	95.24%	1
	MIPO	6	5.26%	3	5.88%	3	4.76%	
Distal fixation	Total distal screws	6.2 (± 1.3)		6.3 (± 1.2)		6.1 (± 1.3)		0.2179
	Bicortical distal screws	4.1 (± 2.2)		4.5 (± 1.7)		3.7 (± 2.5)		**0.0405**
Additional fixation		52	45.61%	28	54.90%	24	38.10%	0.0899
Reoperation	No	101	88.60%	41	80.39%	60	95.24%	**0.0175**
	Mechanical	8	7.02%	5	9.80%	3	4.76%	0.4635
	Septic	5	4.39%	5	9.80%	0	0.00%	**0.0160**

Bold font symbolizes significant results

### Healing rate and complications upon plate osteosynthesis

To enable proper assessment of the healing tendency of plate-treated fractures, only patients with a minimum follow-up period of at least 6 months were examined (Fig. 1; Table 3; Supplementary Table 1). The patients were further divided into two groups: plate only and plate with additional fixation (plate+). The second group included additional fixations devices such as lag screws, plate screws in the working zone, and cerclages as well as combinations of all three. Patients from the plate + group who received a cerclage were evaluated separately in addition. Eighteen of the identified 42 patients (43%) were treated with a plate only, whereas the remaining 24 patients (57%) received an additional fixation. The plate+ and also the cerclage group include a slightly higher amount of A2 (16.67%/22.22% versus 5.56%), A3 (8.33%/22.22% versus 5.56%), and (especially the plate+) also an increased number of C2 fractures (8.33% versus 5.56%) according to the AO classification. Also, more periprosthetic C type (37.50% versus 33.33%) and less periprosthetic B-type fractures (4.17% versus 22.22%) were detected in the plate+ compared with the plate-only group. Patients requiring additional fixation show a higher amount of risk factors as vascular or malignant diseases, nicotine or NSAID consumption, and advanced age. The treatment with plate only led to a union rate of 78% compared with 58% in the plate+ group. Focusing on the number of revision procedures, about one-third of all patients in the plate+ group needed a second intervention, most of them (29%) due to a mechanical reason (broken screws or plate, pseudarthrosis, or similar). Difference of the Parker mobility score of the patients before their trauma and after the treatment showed that more than half of the patients in the plate (56.25%) and the plate+ group (52.63%) regained their original mobility following plate treatment. However, patients treated by plate+ in combination with a cerclage had worse outcome, with only 33,33% reaching their original mobility. Although no significant difference was identified, only 56% of all patients treated with a cerclage showed sufficient union signs at their last clinical follow-up. The revision rate was comparable to the plate+ group, but reduced mobility outcome of patients who received additional cerclage stabilization was found in 66% of these patients.

**Table 3:** Healing and complication rate following plate treatment

		All plates		Plate only		Plate+		*p*-Value	Plate+cerclage		*p*-Value
*n*		42		18	42.86%	24	57.14%		9		
Healing	Union	28	66.67%	14	77.78%	14	58.33%	0.3214	5	55.56%	0.3748
	Nonunion	14	33.33%	4	22.22%	10	41.67%		4	44.44%	
Type of reoperation	No	32	76.19%	16	88.89%	16	66.67%	0.1558	6	66.67%	0.1361
	Mechanical revision	8	19.05%	1	5.56%	7	29.17%		3	33.33%	
	Septic revision	2	4.76%	1	5.56%	1	4.17%		0	0.00%	
Difference in Parker mobility score	Mobility improvement	0	0.00%	0	0.00%	0	0.00%	0.8150	0	0.00%	0.8788
	0	19	45.24%	9	56.25%	10	52.63%		2	33.33%	
	1	4	9.52%	3	18.75%	1	5.26%		1	16.67%	
	2	5	11.90%	1	6.25%	4	21.05%		1	16.67%	
	3	2	4.76%	1	6.25%	1	5.26%		1	16.67%	
	4	3	7.14%	1	6.25%	2	10.53%		0	0.00%	
	5	0	0.00%	0	0.00%	0	0.00%		0	0.00%	
	6	2	4.76%	1	6.25%	1	5.26%		1	16.67%	
	7	0	0.00%	0	0.00%	0	0.00%		0	0.00%	
	8	0	0.00%	0	0.00%	0	0.00%		0	0.00%	
	9	0	0.00%	0	0.00%	0	0.00%		0	0.00%	
	N/a	7	16.67%	2	Valid percentage only	5	Valid percentage only		3	Valid percentage only	

**Fig. 4:**
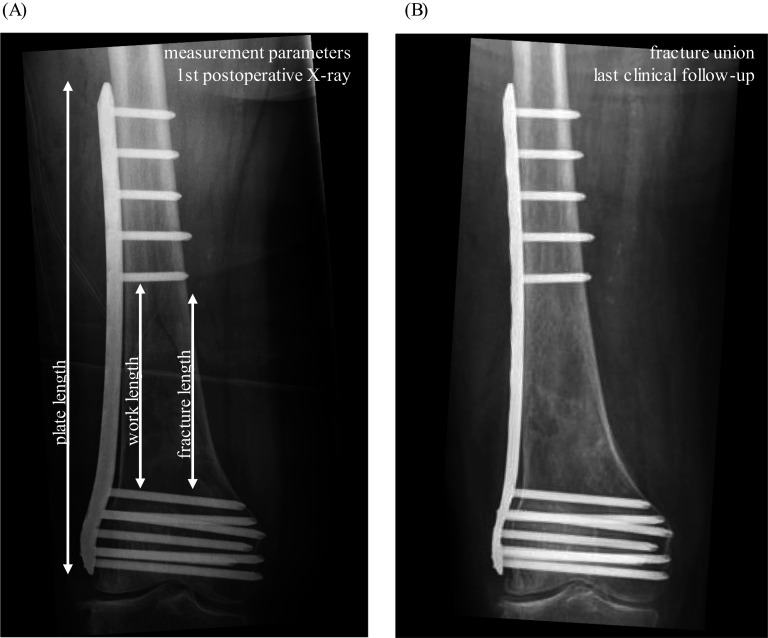
X-ray example: measured parameters and example of fracture union

To further analyze surgical variables, measurement of plate length, work length, and fracture length following plate osteosynthesis was performed (Fig. 3; Table 4). No significant difference was detected when comparing the respective measurements regarding fracture union. Nevertheless, cases with nonunion at final follow-up had a tendency toward comparatively higher plate to fracture lengths. In the plate-only group, a longer work length to fracture length ratio was detected in nonunion cases. Interestingly, the relationship between work length and fracture length appeared to be opposite in plate+cerclage compared with plate only (Fig. 4).

**Table 4:** Plate treatment outcome (plate = plate length; fract = fracture length; work = work length)

		Plates with follow-up > 6 m			
		All patients	Union	Nonunion	*p*-Value
All plates	Plate/fract	3.9 (± 2.6)	3.8 (± 2.3)	4.3 (± 3.1)	0.8336
	Plate/work	3.1 (± 141)	3.2 (± 1.6)	2.8 (± 0.9)	0.3637
	Work/fract	1.3 (± 0.7)	1.3 (± 0.7)	1.4 (± 0.7)	0.4471
Plate only	Plate/fract	4.8 (± 2.8)	4.7 (± 2.9)	4.9 (± 3.0)	0.9510
	Plate/work	3.3 (± 1.1)	3.4 (± 1.1)	2.8 (± 0.7)	0.2280
	Work/fract	1.5 (± 0.8)	1.4 (± 0.7)	1.7 (± 0.9)	0.6017
Plate+	Plate/fract	3.3 (± 2.2)	2.8 (± 0.9)	4.0 (± 3.3)	0.1850
	Plate/work	3.0 (± 1.7)	3.1 (± 2.0)	2.8 (± 1.0)	0.5923
	Work/fract	1.2 (± 0.5)	1.1 (± 0.5)	1.3 (± 0.6)	0.3289
Plate+cerclage	Plate/fract	4.0 (± 3.1)	3.1 (± 0.8)	5.0 (± 4.8)	0.9048
	Plate/work	2.8 (± 1.1)	2.3 (± 0.8)	3.4 (± 1.2)	0.1905
	Work/fract	1.4 (± 0.6)	1.5 (± 0.5)	1.3 (± 0.8)	0.5556

## Discussion

Fractures of the distal femur make up 3–6% of all femoral fractures and in general about < 1% of all human fractures [1, 23]. Despite advances in implant development over the last few years, the complication rate remains comparatively high [12]. The aim of this study is to collect and evaluate patients with fractures of the distal femur between 2015 and 2022 at our level I trauma center and to gather data regarding reoperation and general complications in patients who underwent plate osteosynthesis.

The acquired general demographic verifies the previously described increase of distal femoral fractures in old women [3, 23]. This phenomenon can be explained by the reduced bone quality and increased fall risk in elderly patients [3, 23]. Screening of known risk factors for fractures additionally outlined advanced age and osteoporosis as the two most frequently recorded of all captured risk factors. A simple fall was identified as the most common cause of this fracture. This finding is in line with the previously described high amount of distal femoral fractures after fall at home in elderly patients [23]. Screening of the recorded male cases with regard to their age showed a much more diffuse distribution, with most cases found between 40 and 50 years of age. This result corresponds to published data showing an increased incidence of fractures in younger male patients compared with women [23]. On the basis of available literature, we are confident that our included cohort reflects demographics of patients in daily clinical practice well.

Total knee arthroplasty is a successful surgical intervention in end-stage knee osteoarthritis [24]. Although the best outcome for this treatment was described in patients around 75 years, this intervention is also performed in other age groups including < 55 years and > 90 years [25]. Periprosthetic fractures after total knee arthroplasty are described to appear in about 0.3–2.5% cases after primary and 1.6–38% after revision arthroplasty [2, 26]. A comprehensive study by Court-Brown et al. describes a general incidence of distal femur fractures of about 0.4%, but unfortunately this work did not address the differences between periprosthetic and non-periprosthetic fractures [1]. In our study, a significant difference in age of periprosthetic fractures compared with non-periprosthetic fractures was recognized. A mean age of revisions after arthroplasty of the distal femur was previously described at about 65–74 years, however periprosthetic fractures made up about 4.7% of all indications [27]. Meek et al. reported an increased risk of periprosthetic fractures after arthroplasties in aged female, which goes along with our finding [28]. Our study additionally shows that there is a high likelihood of a periprosthetic fracture after simple fall, compared with other trauma mechanisms, whereas in non-periprosthetic cases, traffic accidents appear to have a significant higher rate of non-periprosthetic fractures. Owing to the lower age of the patients in this group, a stronger bone structure is more likely, which requires significantly more force, as in a traffic accident, to break in the distal femoral region [23]. However, this fact does not explain why there are no cases recorded with present prosthesis. Our study detected a higher reoperation rate owing to septic complications in periprosthetic fractures of the distal femur after fixation by plate, compared with the non-periprosthetic cases. As periprosthetic fractures are typically in frail patients with low bone quality, the treatment of this kind of injuries are challenging to treat [29]. More than 25% of all revisions after arthroplasty are indicated owing to infection [30]. In addition, previous implant surgery is known to have a higher risk for infection, especially in the area of the knee and the shoulder [31]. Therefore it is conceivable that repeated surgery in this area to fix a fracture, which is presumably just as time-consuming as the primary intervention, also carries an increased risk of infection compared with surgery without an implant to address during the operation. Furthermore, the technique of fixation, especially in the distal area of the femur, is far more challenging in case of periprosthetic fractures as the screws need to be placed around the preexisting implant. It is conceivable that repeated drilling to bypass the intramedullary parts of the prosthesis also contributes to a higher risk of infection and additional loosening of the prosthesis.

Our findings indicate a union rate after surgical treatment with plating of distal femur fractures of about 67%, however the cases treated with plates only, without any cerclage or other limitation of the working zone, appear to have a better union rate (78%). In 2017, Koso et al. performed a systematic review and metaanalysis with focus on the healing and reoperation rate after surgical treatment of distal femoral fractures [12]. Interestingly they described a better final healing rate of about 86% after plate treatment compared with our study. The rate of infections upon plate fixation were much closer (3%) to the result described herein (4.7%). Our study found a mechanical failure prevalence of 5.56% in case of plate-only treatment, which also comes closer to the 2–4% reported in the Koso study [12]. A better outcome with less complications seems to result from plate treatment only, while additional fixations (plate+) such as lag screws, intermittent plate screws in the working zone, and/or cerclages seem to result in reduced union and cause more reoperations as well as reduced return to mobility. Sufficient cerclage wiring requires good fracture exposure, dissection, tightening, and tensioning around the broken bone for reduction and stabilization of the fracture [32, 33]. Although a cerclage can provide mechanical fixation, particularly in comminuted cases, there are numerous concerns such as bone necrosis and osteolysis, nonunion, vascular injury, infection, and even mechanical failure [13–17]. Therefore, the results presented here from our center confirm previously described negative effects of cerclage wiring in open reduction and internal fixation treatment. In literature, some previous studies describe no relationship between the usage of cerclage wire and the union rate of femoral fractures [34, 35]. The results presented herein do not support these findings but rather suggest an essential impact of the wire on the union rate and also on the need for subsequent mechanic revision. Screening of the fracture classifications shows a higher usage of additional fixations in more complex fracture situations. This might be reasonable in specific cases, but since our findings depict sufficient treatment also of more complex fractures by plate only with a lower risk of complications and nonunion, the indication for additional fixations, especially of cerclage wiring, needs to be very strict as the additional benefit is doubtful.

Locking plate fixation is the method most commonly performed for surgical stabilization of distal femoral fractures as it brings biomechanical stabilization, especially against torsional loading [36]. Various studies describe successful results such as union rates between 85% and 100% and fixation maintenance [37–40]. Multiple studies discuss the relevance of an appropriate working zone and describe problems in union and lack of callus formation due to too rigid fixation by plate [22, 41–43]. Our study revealed no significant differences when comparing the union rate of distinct plate treatments with focus on plate length, work length, fracture length, and their relations. However, in 2011, Henderson et al. investigated 86 cases of distal femoral fractures after surgical treatment using locking plates. They analyzed the relationship between plate length, working zone, and bone union without any significant results [21]. Comparing the working zone to plate length ratio between union and nonunion cases did not even identify a trend interpretation in their work. Stoffel et al. described the relevance of three screws on either side of the fracture as necessary for a sufficient stabilization, however a comminuted fracture requires a stiffer fixation, with closer screw placement, than simple fractures of the lower extremity [44]. Leaving an empty hole on both sides of the fracture on the other side reduces the stiffness by about 50% [44]. Another study reported that the relationship between stiffness and working length depends on the plate material [18]. Studies from Lujan et al. and from Harvin et al. described no significant impact of the working length on the fracture healing [22, 45]. Therefore, the impact of the plate length and the work length in relation to the fracture length appears to depend on as yet not included factors to achieve a higher guarantee of more sufficient bone healing.

Our study has some apparent limitations. As a retrospective study conducted at one center, it describes a heterogeneous patient population in terms of patient data, fracture patterns, and plate–screw constructions. The operations were carried out by different surgeons, which entailed personal influencing factors such as surgical ability, the choice of plates, individual screw placement, and varying follow-up treatments. Different plates were used for treatment; however, they were all suitable for the treatment of distal femoral fractures. The varying plate parameters, whether owing to the choice of plate by the surgeon or the plate design, probably also affect the complication rate as well as the healing tendency after treatment. The number of patients is also a clear limitation in terms of the significance of this study, which could probably be compensated subsequently by including data from additional trauma centers.

## Conclusions

The present study provides a comprehensive overview of the occurrence and outcome of distal femur fractures treated in a level 1 trauma center. The sex-specific frequency of distal femoral fractures was verified, and key surgical variables and their influence on union and functional outcome following plate osteosynthesis were described. The detected periprosthetic fractures of the distal femur treated by plate revealed a significantly higher complication rate, especially due to septic events, compared with non-periprosthetic fractures. Usage of cerclage wires in the fractures area was associated with higher complication and nonunion rates.

## Supplementary Information

Additional file 1.

## Data Availability

The datasets used and/or analyzed during the current study are available from the corresponding author on reasonable request.
