# Supplementary material for: Distal femoral fractures: periprosthetic fractures have four times more complications than non-periprosthetic fractures and cerclage should be avoided: retrospective analysis of 206 patients
Source: J Orthop Traumatol. 2024 Sep 3;25:41. doi: 10.1186/s10195-024-00782-2 (PMC11371989; doi:10.1186/s10195-024-00782-2)
Supplement: Supplementary file 1 — Additional file 1. [file 10195_2024_782_MOESM1_ESM.docx]

Supplementary Table 1. Healing and complication rate following plate treatment – additional information

|  |  | all plates | | plate only | | plate + | |  | plate + cerclage | |
| --- | --- | --- | --- | --- | --- | --- | --- | --- | --- | --- |
| n | | 42 | | 18 | 42,86% | 24 | 57,14% |  | 9 | |
| Mean age (y) | | 76 (±14) | | 80 (±13) | | 74 (±15) | |  | 80 (±8) | |
| Female (%) | | 37 | 88,10% | 17 | 94,44% | 20 | 83,33% |  | 8 | 88,89% |
| AO classification  (non-periprosthetic) | A1 | 7 | 16,67% | 3 | 16,67% | 4 | 16,67% |  | 1 | 11,11% |
|  | A2 | 5 | 11,90% | 1 | 5,56% | 4 | 16,67% |  | 2 | 22,22% |
|  | A3 | 3 | 7,14% | 1 | 5,56% | 2 | 8,33% |  | 2 | 22,22% |
|  | B1 | 0 | 0,00% | 0 | 0,00% | 0 | 0,00% |  | 0 | 0,00% |
|  | B2 | 0 | 0,00% | 0 | 0,00% | 0 | 0,00% |  | 0 | 0,00% |
|  | B3 | 0 | 0,00% | 0 | 0,00% | 0 | 0,00% |  | 0 | 0,00% |
|  | C1 | 0 | 0,00% | 0 | 0,00% | 0 | 0,00% |  | 0 | 0,00% |
|  | C2 | 3 | 7,14% | 1 | 5,56% | 2 | 8,33% |  | 0 | 0,00% |
|  | C3 | 2 | 4,76% | 1 | 5,56% | 1 | 4,17% |  | 0 | 0,00% |
| UCS classification  (periprosthetic) | A | 0 | 0,00% | 0 | 0,00% | 0 | 0,00% |  | 0 | 0,00% |
|  | B | 5 | 11,90% | 4 | 22,22% | 1 | 4,17% |  | 0 | 0,00% |
|  | C | 15 | 35,71% | 6 | 33,33% | 9 | 37,50% |  | 3 | 33,33% |
|  | D | 2 | 4,76% | 1 | 5,56% | 1 | 4,17% |  | 1 | 11,11% |
|  | E | 0 | 0,00% | 0 | 0,00% | 0 | 0,00% |  | 0 | 0,00% |
|  | F | 0 | 0,00% | 0 | 0,00% | 0 | 0,00% |  | 0 | 0,00% |
| nicotine | yes | 5 | 16,13% | 0 | 0,00% | 5 | 27,78% |  | 0 | 0,00% |
|  | no | 26 | 83,87% | 13 | 100,00% | 13 | 72,22% |  | 7 | 100,00% |
|  | n.a. | 11 | valid percentage only | 5 | valid percentage only | 6 | valid percentage only |  | 2 | valid percentage only |
| diabetes | yes | 5 | 16,13% | 6 | 33,33% | 3 | 13,64% |  | 1 | 11,11% |
|  | no | 26 | 83,87% | 12 | 66,67% | 19 | 86,36% |  | 8 | 88,89% |
|  | n.a. | 11 | valid percentage only | 0 | valid percentage only | 2 | valid percentage only |  | 0 | valid percentage only |
| vascular disease | yes | 9 | 22,50% | 3 | 16,67% | 4 | 18,18% |  | 2 | 22,22% |
|  | no | 31 | 77,50% | 15 | 83,33% | 18 | 81,82% |  | 7 | 77,78% |
|  | n.a. | 2 | valid percentage only | 0 | valid percentage only | 2 | valid percentage only |  | 0 | valid percentage only |
| advanced age | yes | 7 | 17,50% | 15 | 83,33% | 22 | 91,67% |  | 9 | 100,00% |
|  | no | 33 | 82,50% | 3 | 16,67% | 2 | 8,33% |  | 0 | 0,00% |
|  | n.a. | 2 | valid percentage only | 0 | valid percentage only | 0 | valid percentage only |  | 0 | valid percentage only |
| malignant disease | yes | 37 | 88,10% | 2 | 11,11% | 5 | 20,83% |  | 2 | 22,22% |
|  | no | 5 | 11,90% | 16 | 88,89% | 19 | 79,17% |  | 7 | 77,78% |
|  | n.a. | 0 | valid percentage only | 0 | valid percentage only | 0 | valid percentage only |  | 0 | valid percentage only |
| rheumatism | yes | 7 | 16,67% | 0 | 0,00% | 0 | 0,00% |  | 0 | 0,00% |
|  | no | 35 | 83,33% | 18 | 100,00% | 24 | 100,00% |  | 9 | 100,00% |
|  | n.a. | 0 | valid percentage only | 0 | valid percentage only | 0 | valid percentage only |  | 0 | valid percentage only |
| nsaid | yes | 0 | 0,00% | 2 | 12,50% | 3 | 13,64% |  | 2 | 25,00% |
|  | no | 42 | 100,00% | 14 | 87,50% | 19 | 86,36% |  | 6 | 75,00% |
|  | n.a. | 0 | valid percentage only | 2 | valid percentage only | 2 | valid percentage only |  | 1 | valid percentage only |
| steroid use | yes | 5 | 13,16% | 1 | 6,25% | 1 | 4,55% |  | 0 | 0,00% |
|  | no | 33 | 86,84% | 15 | 93,75% | 21 | 95,45% |  | 8 | 100,00% |
|  | n.a. | 4 | valid percentage only | 2 | valid percentage only | 2 | valid percentage only |  | 1 | valid percentage only |
| osteoporosis | yes | 2 | 5,26% | 5 | 31,25% | 5 | 20,83% |  | 2 | 22,22% |
|  | no | 36 | 94,74% | 11 | 68,75% | 19 | 79,17% |  | 7 | 77,78% |
|  | n.a. | 4 | valid percentage only | 2 | valid percentage only | 0 | valid percentage only |  | 0 | valid percentage only |
